# Tailoring TiO_2_ Nanotube‐Interlaced Graphite Carbon Nitride Nanosheets for Improving Visible‐Light‐Driven Photocatalytic Performance

**DOI:** 10.1002/advs.201700844

**Published:** 2018-04-15

**Authors:** Yang Wang, Xueqin Liu, Cunchuan Zheng, Yinchang Li, Songru Jia, Zhen Li, Yanli Zhao

**Affiliations:** ^1^ Faculty of Materials Science and chemistry China University of Geosciences Wuhan 430074 P. R. China; ^2^ Division of Chemistry and Biological Chemistry School of Physical and Mathematical Sciences Nanyang Technological University 21 Nanyang Link Singapore 637371 Singapore

**Keywords:** graphite carbon nitride, photocatalysis, template‐tailoring methods, TiO_2_ nanotubes, visible light

## Abstract

Rapid recombination of photoinduced electron–hole pairs is one of the major defects in graphitic carbon nitride (g‐C_3_N_4_)‐based photocatalysts. To address this issue, perforated ultralong TiO_2_ nanotube‐interlaced g‐C_3_N_4_ nanosheets (PGCN/TNTs) are prepared via a template‐based process by treating g‐C_3_N_4_ and TiO_2_ nanotubes polymerized hybrids in alkali solution. Shortened migration distance of charge transfer is achieved from perforated PGCN/TNTs on account of cutting redundant g‐C_3_N_4_ nanosheets, leading to subdued electron–hole recombination. When PGCN/TNTs are employed as photocatalysts for H_2_ generation, their in‐plane holes and high hydrophilicity accelerate cross‐plane diffusion to dramatically promote the photocatalytic reaction in kinetics and supply plentiful catalytic active centers. By having these unique features, PGCN/TNTs exhibit superb visible‐light H_2_‐generation activity of 1364 µmol h^−1^ g^−1^ (λ > 400 nm) and a notable quantum yield of 6.32% at 420 nm, which are much higher than that of bulk g‐C_3_N_4_ photocatalysts. This study demonstrates an ingenious design to weaken the electron recombination in g‐C_3_N_4_ for significantly enhancing its photocatalytic capability.

Semiconductor photocatalysis as a type of green technology for solar energy conversion has exhibited fascinating application prospects in environmental and energy‐relevant areas, such as photocatalytic degradation of pollutants and photocatalytic hydrogen generation.^1^ In the wave of photocatalysis research, graphitic carbon nitride (g‐C_3_N_4_)‐based photocatalysts have been significantly flourishing^2^ since its promising utilization for water splitting under visible‐light irradiation reported in 2009.^3^ As a new generation of metal‐free photocatalysts, g‐C_3_N_4_ exhibits various extraordinary features, such as desirable visible‐light response (λ < 460 nm) with medium bandgap (2.7 eV), environmental friendliness, reasonable cost, and high stability.^4^ However, its photocatalytic performance is severely hampered by high recombination rate of photoinduced electron–hole pairs, low specific surface area, and limited active sites.^5^ Thus, extensive research has been carried out to enhance its photocatalytic activity.^6^ To date, the most common approaches to improve the photocatalytic activity of g‐C_3_N_4_ include doping for narrowing the bandgap,^7^ morphology engineering for light harvesting,^8^ and composite structure control for effective charge separation.[[qv: 1b,9]]

Inspired by the graphene research,^10^ the exfoliation of layered bulk materials into 2D counterparts induces unique physicochemical properties including increased surface area, ultrahigh charge carrier mobility, and pronounced changes in the energy band structure. Indeed, reducing the dimensionality of bulk g‐C_3_N_4_ into 2D nanosheets could increase the surface area that is beneficial to the improvement of photocatalytic efficiency via efficient exposure of active sites, optimized light harvest, and charge separation.^11^ Moreover, lacunaris materials are highly appreciated as catalysts due to their accessible porous nature with a large surface area for the mass transfer.^12^ Hence, porous 2D g‐C_3_N_4_ nanosheets have been prepared to show photocatalytic performance enhancement.^13^

In addition to the nanostructure design, the development of g‐C_3_N_4_ hybrids for photocatalysis is another promising approach.^14^ Among various routes, the introduction of an appropriate band structure at the heterojunction interface is the most important prerequisite to enhance the charge separation efficiency for increasing photocatalytic performance.[[qv: 9a,12a,15]] Since TiO_2_ has an advantage in electron–hole separation for its special energy band,^16^ the establishment of heterostructure between g‐C_3_N_4_ and TiO_2_ is an effective way to improve the efficiency of charge separation in g‐C_3_N_4_/TiO_2_ photocatalyst systems.^17^ 1D TiO_2_ nanotubes having the advantage of intimate contact with 2D nanosheets and cross‐linked network^18^ present a significant potential as photoinduced electron sink in g‐C_3_N_4_/TiO_2_ heterojunction hybrids for the electron–hole separation.

Herein, by utilizing the mixture of ultralong TiO_2_ nanotubes (Figure S1, Supporting Information) and melamine as precursors for the polymerization, we developed a template‐based strategy to generate the perforated ultralong TiO_2_ nanotube‐interlaced g‐C_3_N_4_ nanosheets (PGCN/TNTs) having in situ formed in‐plane holes. As compared to intact ultralong TiO_2_ nanotube‐interlaced g‐C_3_N_4_ nanosheets (GCN/TNTs), the as‐prepared PGCN/TNTs with in‐plane holes can improve the electron–hole separation and solar energy utilization. Meanwhile, the surface hydrophilicity of PGCN/TNTs was well optimized via the alkaline solution treatment, resulting in greater H_2_O affinity. Accordingly, PGCN/TNTs were expected to display high visible‐light photocatalytic activity in H_2_‐generation and dye degradation.

At the initial stage of the preparation reaction, melamine was fully covered on the surface of TiO_2_ nanotubes due to the formation of hydrogen bonds after ultrasonic treatment. Then, the hybrid thermally polymerized into bulk g‐C_3_N_4_/TNTs. In the bulk g‐C_3_N_4_/TNTs, TNTs as the skeleton were implanted into the 2D framework of g‐C_3_N_4_. Under the tailoring treatment of alkali ions^19^ with embedded TNTs as the template, PGCN/TNTs were obtained (**Figure** **1**a). To verify this synthetic process, the hydrolysis was first studied by scanning electron microscope (SEM) and transmission electronic microscope (TEM). Figure 1b shows that pristine bulk g‐C_3_N_4_/TNTs were composited of stacked lamellar textures with sizes around micrometers, and TNTs were not found in the bulk material on account of possible embedment by 2D g‐C_3_N_4_ nanosheets during the copolymerization process. Figure 1c presents the TEM image of the GCN/TNTs. It was found that the nanotubes interlaced with the 2D nanosheets without holes. After tailoring process, holes were distributed uniformly on the micrometers sized PGCN/TNTs (Figure 1d). It was clearly observed from the TEM images (Figure 1e,f) that the exfoliated nanosheets were enriched with holes of ≈100 nm in diameter. The ultralong nanotubes homogeneously interlaced with the retained g‐C_3_N_4_ nanosheets, and no naked nanotubes were observed in holes or edges of nanosheets. Figure 1g shows high‐resolution TEM (HRTEM) image of PGCN/TNTs. Interestingly, an apparent heterointerface was observed between TNTs and g‐C_3_N_4_ nanosheets. Two sets of lattice fringes with vertical interplane spacing of 0.24 and 0.19 nm in TNTs are attributed to (001) and (100) planes of the anatase TiO_2_ phase respectively, and the heterointerface is roughly along the [100] direction of the anatase crystal. In addition, the tailoring function of alkali ion on g‐C_3_N_4_ was also proven by microscopic images (Figure S2, Supporting Information). It was found that without TNTs as the template, the g‐C_3_N_4_ nanosheets were cut into nanofibers. Clearly, the 2D g‐C_3_N_4_ framework was restricted when they contacted with TiO_2_ nanotubes, indicating the template effect of the interlaced TiO_2_ nanotubes. Elemental mapping images from the selected areas of PGCN/TNTs show a homogenous distribution of TiO_2_ nanotubes (Figure 1h).

**Figure 1:**
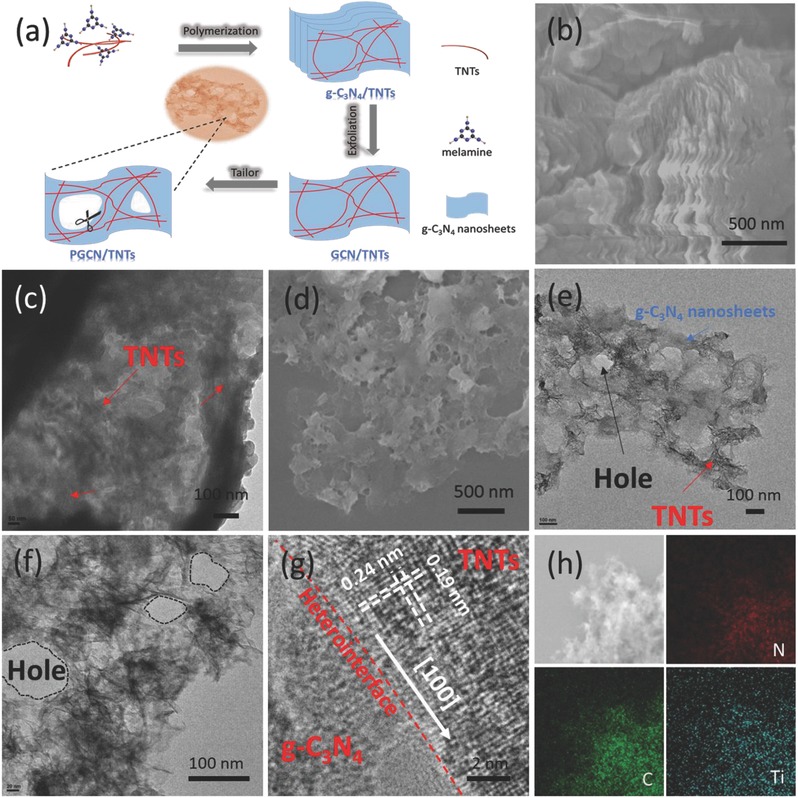
a) Schematic illustration of template‐based alkali ion tailoring strategy from bulk g‐C_3_N_4_/TNTs to PGCN/TNTs. b) SEM image of bulk g‐C_3_N_4_/TNTs. c) TEM image of GCN/TNTs. d–f) SEM and TEM images of PGCN/TNTs. g) HRTEM image of PGCN/TNTs heterostructure. h) SEM image of PGCN/TNTs and corresponding elemental mapping images of nitrogen, carbon, and titanium, indicating homogenous distribution of TNTs over entire PGCN/TNTs.

The crystal structures of the as‐prepared PGCN/TNTs were analyzed by powder X‐ray diffraction (XRD), as shown in **Figure** **2**a. It is well known that the two dominant diffraction peaks at 13.0° and 27.3° in the powder XRD pattern of g‐C_3_N_4_ are associated with the in‐plane trigonal N linkage of tri‐s‐triazine motif and the periodic stacking of layers for conjugated aromatic systems, respectively. The peak located at 25.3° is ascribed to (101) facet of the anatase TiO_2_ (JCPDS no. 89–4921). As compared with bulk g‐C_3_N_4_ and g‐C_3_N_4_/TNTs, the decreased intensity of peak at 27.3° in GCN/TNTs and PGCN/TNTs verified that the layered g‐C_3_N_4_ structure was successfully exfoliated into nanosheets. Almost disappeared peak of PGCN/TNTs at 13.0° (Figure S3, Supporting Information) is mainly due to the fact that the alkali ion interaction with the in‐plane trigonal nitrogen during prolonged tailoring process for the formation of in‐plane holes may decrease the ordering degree of in‐plane structures.

**Figure 2:**
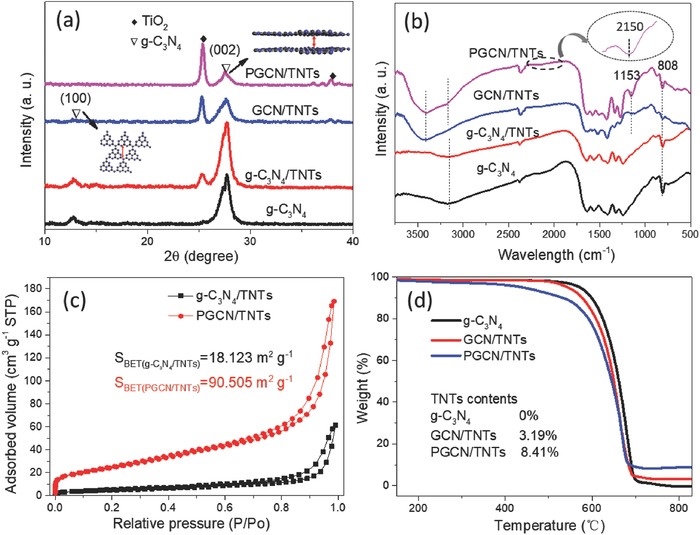
a) Powder XRD patterns and b) FTIR spectra of g‐C_3_N_4_, g‐C_3_N_4_/TNTs, GCN/TNTs, and PGCN/TNTs. c) N_2_ adsorption/desorption isotherms of bulk g‐C_3_N_4_/TNTs and PGCN/TNTs. d) TGA profiles of g‐C_3_N_4_, GCN/TNTs, and PGCN/TNTs.

The chemical structures of g‐C_3_N_4_, g‐C_3_N_4_/TNTs, GCN/TNTs, and PGCN/TNTs were studied by Fourier transform infrared (FTIR) spectra as shown in Figure 2b. All of the bands attributable to a typical g‐C_3_N_4_ were observed, further demonstrating that the chemical structure of tri‐s‐triazine ring unit is robust against ultrasonic and alkali treatment. Specifically, broad peaks between 3000 and 3500 cm^−1^ are assigned to amine group and water. Compared with bulk g‐C_3_N_4_ and g‐C_3_N_4_/TNTs, peaks of PGCN/TNTs between 3000 and 3500 cm^−1^ became stronger, suggesting higher exposure of surface amino (N—H) and hydroxyl (O—H) groups in PGCN/TNTs. The presence of surface amino and hydroxyl groups indicates high exposure edges and surfaces in PGCN/TNTs, which is consistent with the direct observation by TEM. When compared to GCN/TNTs, it was noted that a new sharp peak at 1153 cm^−1^ appeared in the FTIR spectrum of PGCN/TNTs. This peak is ascribed to the vibration of C—O—C group,^20^ indicating that O atoms are preferentially bonded to C atoms by substituting coordinated N atoms. The O doping could optimize the band structure of g‐C_3_N_4_,^16^ leading to the light absorption increase of PGCN/TNTs in the visible region (Figure S4, Supporting Information). When compared with g‐C_3_N_4_ and g‐C_3_N_4_/TNTs, the FTIR spectrum of PGCN/TNTs displays an additional peak at around 2150 cm^−1^, which can be assigned to the vibration of cyano terminal group (C≡N).[[qv: 7a]] The presence of C≡N proves that the tri‐s‐triazine ring was partly fractured and restructured for atomic substitution during ultraphonic tailoring treatment.

Brunauer–Emmett–Teller (BET) surface areas of the as‐prepared samples were determined by N_2_ adsorption/desorption measurements at 77.4 K. As shown in Figure 2c and Figure S5 (Supporting Information) (pore size distributions), PGCN/TNTs exhibit a large BET surface area of 90.505 m^2^ g^−1^, which is almost four times higher than that of g‐C_3_N_4_/TNTs (18.123 m^2^ g^−1^), indicating efficient exfoliation of PGCN/TNTs. Significantly, high‐specific surface area can present more active sites for the adsorption and surface reaction. The quantitative compositional analysis of TiO_2_ content in different composites was conducted by thermal gravimetric analysis (TGA) as shown in Figure 2d. The main weight loss appears at the range of 500−700 °C due to the combustion of g‐C_3_N_4_. The total weight loss is 100% for pure g‐C_3_N_4_, implying that it decomposes completely when the temperature reaches 700 °C. The retained weight is the weight percent of TiO_2_ in the composites. The amount of TiO_2_ in the composites was measured to be 3.19 and 8.41 wt% for GCN/TNTs and PGCN/TNTs, respectively. As compared to GCN/TNTs, higher TiO_2_ content in PGCN/TNTs is due to the tailoring treatment of g‐C_3_N_4_ by the alkaline solution for the hole formation.

The compositions of surface elements and their oxidation states in PGCN/TNTs, together with the interaction between TiO_2_ nanotubes and g‐C_3_N_4_ were analyzed by X‐ray photoelectron spectroscopy (XPS). The XPS survey spectra (Figure S6, Supporting Information) confirm that all g‐C_3_N_4_‐based samples are mainly composed of carbon, nitrogen, and oxygen, and g‐C_3_N_4_/TNTs and PGCN/TNTs contain some titanium. The strong peak (**Figure** **3**a) pertaining to N 1s (398.3 eV) from g‐C_3_N_4_ is assigned to sp^2^ hybridized N (C—N=C).[[qv: 8a]] The peaks in N 1s region at around 399.3 and 401.2 eV are assigned to ternary nitrogen (N—C3) and C—N—H groups, respectively.[[qv: 5a]] As compared with g‐C_3_N_4_, PGCN/TNTs show unaltered functional groups, indicating stable graphite carbon–nitrogen structure in PGCN/TNTs. Interestingly, N 1s core level spectrum of PGCN/TNTs shifts to higher binding energies by 0.4 eV, implying the interface formation between TiO_2_ nanotubes and g‐C_3_N_4_ in PGCN/TNTs. Figure 3b shows the binding energy region of C 1s with the C=C peak located at 284.6 eV. Another peak observed at 288.1 eV in this region is related to sp^2^‐hybridized carbon (N—C=N) from g‐C_3_N_4_. A new peak at 286.1 eV observed in PGCN/TNTs is assigned to C—O species. These results suggest that there are some C—O—H groups on the surface of PGCN/TNTs after the ultrasonic/alkali ion treatment. The high‐resolution O 1s peak (Figure 3c) of g‐C_3_N_4_/TNTs at around 529.42 eV is associated with the Ti—O bond in TiO_2_ nanotubes. After the hydrolyzing process of g‐C_3_N_4_/TNTs, the peak at 531.58 eV representing hydroxyl (O—H) group increases dramatically. The deconvoluted spectra of C and O essentially indicate that layered bulk g‐C_3_N_4_/TNTs were successfully disintegrated with functional groups such as —OH at surfaces under alkaline condition, which would be beneficial for the dispersion of PGCN/TNTs in aqueous solution (Figure S7, Supporting Information). The peaks in the Ti 2p spectrum of PGCN/TNTs display a shift of 0.8 eV to lower binding energy as compared with that of TNTs (Figure 3d), further confirming a synergistic effect between g‐C_3_N_4_ and TiO_2_ in PGCN/TNTs.

**Figure 3:**
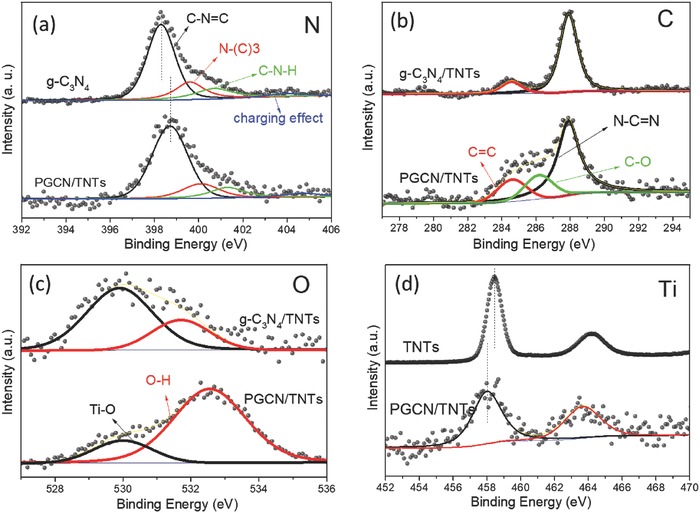
XPS spectra. a) N 1s region of g‐C_3_N_4_ and PGCN/TNTs. b) C 1s and c) O 1s regions of g‐C_3_N_4_/TNTs and PGCN/TNTs. d) Ti 2p region of TNTs and PGCN/TNTs.

The photocatalytic performance of PGCN/TNTs was evaluated by hydrogen production and dye degradation under visible‐light irradiation. The H_2_ production activities were compared among bulk g‐C_3_N_4_, g‐C_3_N_4_/TNTs, GCN/TNTs, and PGCN/TNTs in **Figure** **4**a,b. g‐C_3_N_4_ exhibits a rather low H_2_‐generation rate of 6.85 µmol h^−1^, arising from its poor charge transport capability and low‐specific surface area, while the g‐C_3_N_4_/TiO_2_ hybrids show an apparent enhancement in the H_2_‐generation rate, primarily due to effectively constructed heterojunction for the electron–hole separation. Specially, PGCN/TNTs show the highest H_2_ evolution rate of 68.20 µmol h^−1^, which is 9.0, 3.9, and 0.6 times higher than that of g‐C_3_N_4_, g‐C_3_N_4_/TNTs, and GCN/TNTs, respectively. The excellent H_2_‐generation performance of PGCN/TNTs makes it one of leading materials with high H_2_ production rate in the reported melamine‐based g‐C_3_N_4_ photocatalysts (Figure S8, Supporting Information). The photocatalytic H_2_ evolution stability of PGCN/TNTs was confirmed by cycling experiments. As shown in Figure 4a, the hydrogen evolution activity of PGCN/TNTs was maintained without noticeable deactivation of photocatalytic performance in four cycles for 20 h, indicating good stability of the photocatalyst. The apparent quantum efficiency (AQE) versus the wavelength of the incident light was exhibited in Figure 4c, suggesting that AQE at 420 nm could reach up to 6.32%. Interestingly, the AQE of PGCN/TNTs remains 1.04% at 500 nm, which is higher than most of other g‐C_3_N_4_‐based materials (Table S1, Supporting Information). In sharp contrast, the quantum efficiency of pristine g‐C_3_N_4_ is much lower in both the UV and visible‐light regions (0.86% at 420 nm). Therefore, these results demonstrate PGCN/TNTs as promising and efficient photocatalysts possess excellent quantum efficiency. In addition, the photocatalytic degradation of rhodamine B (RhB) was also measured under visible‐light irradiation (λ > 400 nm). As presented in Figure S9 (Supporting Information), the degradation rate of PGCN/TNTs (0.051 min^−1^) for RhB is about 3.6 and 12.8 times higher than that of GCN/TNTs and g‐C_3_N_4_/TNTs respectively, which is consistent with the photocatalytic activity of H_2_ evolution.

**Figure 4:**
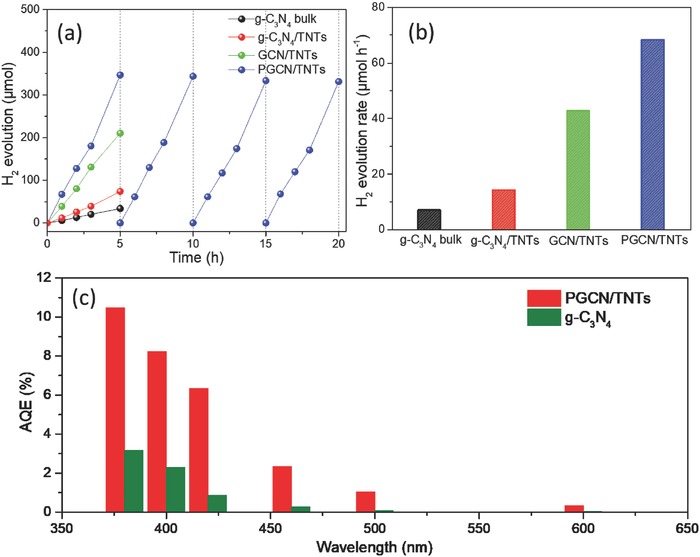
Time courses of water splitting for a) H_2_ production and b) photocatalytic H_2_ production rate under visible light (>400 nm) using as‐synthesized g‐C_3_N_4_, g‐C_3_N_4_/TNTs, GCN/TNTs, and PGCN/TNTs as photocatalysts. c) AQE against light wavelength of PGCN/TNTs and g‐C_3_N_4_.

For gaining the insights into the photocatalysis mechanism, photoelectrochemical properties of the prepared samples were also investigated. The photoelectrochemical properties of g‐C_3_N_4_/TiO_2_ hybrids before and after the tailoring process were investigated by measuring their transient photocurrent response and electrochemical impedance responses. As shown in **Figure** **5**a, all of the samples exhibit rapid photocurrent responses upon the light is turned on and off, and the responses remain at a relatively stable value during the irradiation process. Such phenomenon was well repeated in each on–off cycle, indicating good reproducibility of the samples. Further observation shows that the photocurrent of the g‐C_3_N_4_/TNTs electrode (0.01 mA cm^−2^) is doubled as compared to that of g‐C_3_N_4_ (0.005 mA cm^−2^), demonstrating that the separation efficiency of the photoinduced electron–hole pairs in g‐C_3_N_4_/TNTs is improved due to unique interfacial charge transfer of heterojunction. As compared to bulk g‐C_3_N_4_/TNTs, GCN/TNTs exhibit higher photocurrent intensity, as the exfoliated nanosheet structure exposes more active sites to restrain the recombination.^21^ PGCN/TNTs show the highest photocurrent density (0.049 mA cm^−2^), demonstrating that the charge carrier separation in tailored nanostructure of PGCN/TNTs is further improved. A similar tendency was obtained in the electrochemical impedance spectrum (EIS), as shown in Figure 5b. By fitting the obtained impedance spectra with the transmission line model (Figure S10, Supporting Information), the parameters including series resistance (*R*1) and electron transport resistance (*R*2) were obtained (Table S2, Supporting Information). The arc radius of PGCN/TNTs on EIS Nyquist plot is smaller than that of GCN/TNTs and g‐C_3_N_4_, indicating that PGCN/TNTs have the lowest *R*2 value (1.04 × 10^5^ Ω), enabling fast interfacial charge carrier transfer. These results further reveal that faster interfacial charge migration and more effective separation of charge carriers on the PGCN/TNTs surface are responsible for enhanced photocatalytic activity.

**Figure 5:**
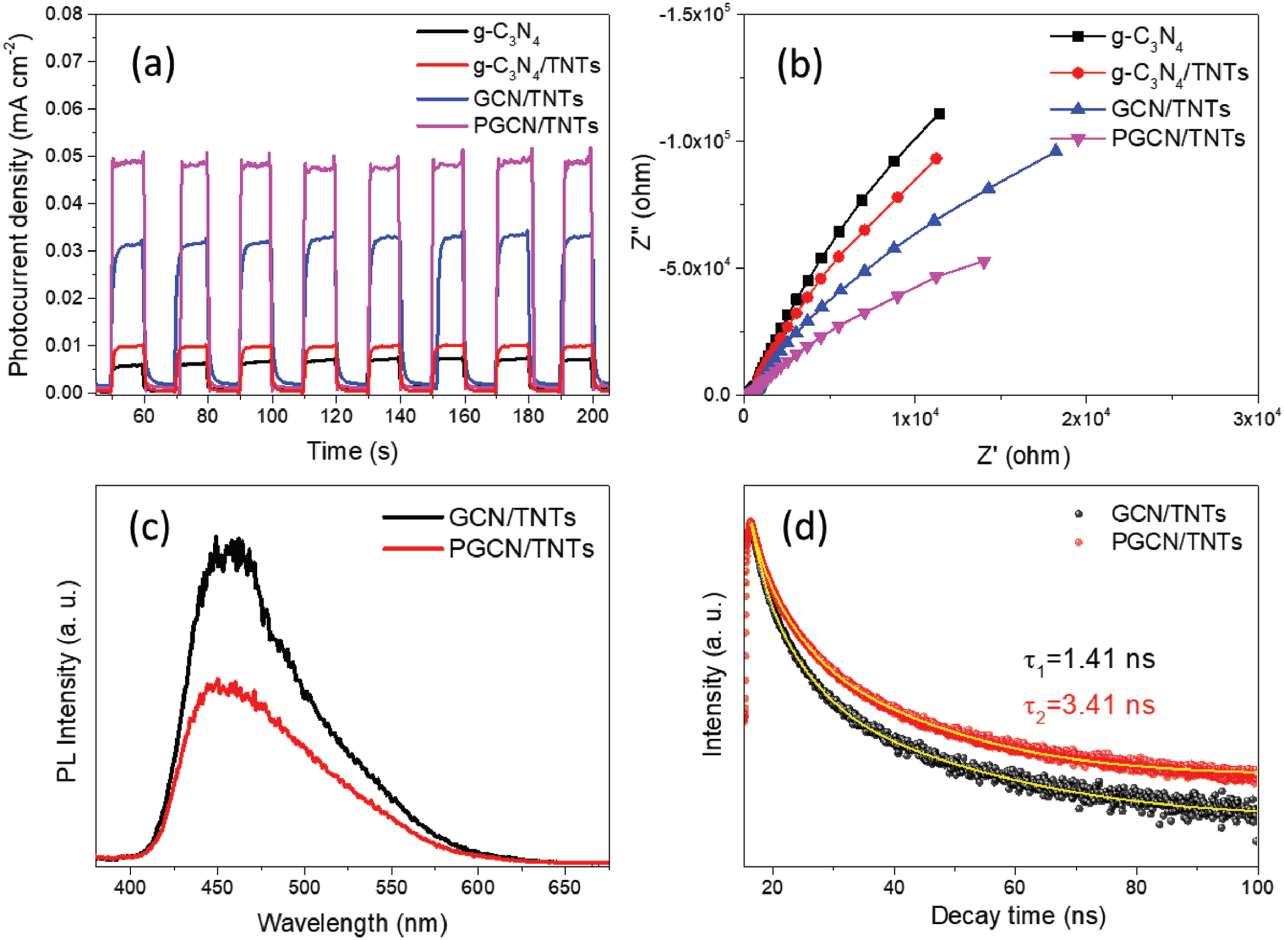
a) Transient photocurrent response and b) electrochemical impedance spectra of g‐C_3_N_4_, g‐C_3_N_4_/TNTs, GCN/TNTs, and PGCN/TNTs. c) PL spectra and d) time‐resolved transient PL spectra of GCN/TNTs and PGCN/TNTs.

The effect of nanostructure tailoring on the carrier recombination in PGCN/TNTs was then studied by photoluminescence (PL) spectroscopy. g‐C_3_N_4_ exhibits the strongest PL with a peak emission at about 460 nm, which is in good agreement with the analysis of UV–vis absorbance spectra (Figure S4, Supporting Information). As compared with GCN/TNTs, PGCN/TNTs show a reduced recombination loss of photogenerated electron–hole pairs by the efficient charge transfer. Some studies showed that redundant g‐C_3_N_4_ nanosheets of GCN/TNTs would be the electron–hole recombination center.^14^ Herein, the shortened electron transfer distance (from g‐C_3_N_4_ to g‐C_3_N_4_/TNTs interface) after the tailoring treatment was obtained for effective electron–hole separation in the case of PGCN/TNTs.

Hereafter, the photophysical characteristics of the photogenerated charge carriers for these samples were carried out using time‐resolved transient PL spectra (Figure 5d). A triexponential functions fitting was employed to analyze the PL decay curves, and the average PL lifetime (*τ_n_*) was deduced by the following equation

(1) $$\tau_{n}=\frac{A_{1}\tau_{1}^{2}+A_{2}\tau_{2}^{2}+A_{3}\tau_{3}^{2}}{A_{1}\tau_{1}+A_{2}\tau_{2}+A_{3}\tau_{3}}$$

where *τ_i_* is the estimated lifetime value and *A_i_* is the corresponding amplitude. The resulted average lifetime is 3.41 ns for PGCN/TNTs, which is 1.42 times longer than that of GCN/TNTs. The long average lifetime of PGCN/TNTs means that the recombination of the electron–hole pairs is significantly refrained and greatly depended on the trimming of excessive g‐C_3_N_4_ nanosheets in PGCN/TNTs. Moreover, PGCN/TNTs show electron diffusion length (*L_n_*) of 3.9 times higher than that of GCN/TNTs (Figure S11, Supporting Information). The longer *L_n_* of PGCN/TNTs indicates reduced electron traps during the electron transferring.^22^ By combining these results, it was rationally proposed that the electron transfer pathway of PGCN/TNTs was greatly optimized by the template‐based alkali ion tailoring strategy, leading to efficient photoexcited electron−hole separation and long photocarrier lifetime.

On the basis of the above analysis, the photocatalytic mechanism of PGCN/TNTs was suggested as shown in **Figure** **6**. First, in the synthesized PGCN/TNTs, the heterojunction was formed in matched energy band between g‐C_3_N_4_ nanosheets and TiO_2_ nanotubes to separate photoinduced electron–hole of g‐C_3_N_4_ under visible light. According to the conduction band (CB) and valence band (VB) edge potentials of g‐C_3_N_4_ (−1.12 and 1.57 eV) and TiO_2_ (−0.29 and 2.91 eV), g‐C_3_N_4_ in g‐C_3_N_4_/TiO_2_ could easily absorb the visible light. Once they are irradiated under visible light (>400 nm), electrons are able to jump from the VB to the CB of g‐C_3_N_4_ and then transfer from the CB of g‐C_3_N_4_ nanosheets to that of anchored TiO_2_ nanotubes. Such electron transition between heterojunctions efficiently reduces the recombination of charge carriers and prolongs the charge lifetime. Second, a plenty of electron–hole recombination in g‐C_3_N_4_ nanosheets was eliminated through tailoring redundant g‐C_3_N_4_ nanosheets in PGCN/TNTs. Due to intrinsic features of easy photoinduced electron–hole recombination, g‐C_3_N_4_ nanosheets show a short electron diffusion length. Electrons are induced and consumed by g‐C_3_N_4_ nanosheets at a distance away from the heterojunction interface of GCN/TNTs. After template‐tailoring strategy, the retained PGCN/TNTs could minimize the electron transport path and more solar energy could be utilized, leading to weaker electron–hole recombination and higher photogenerated electron yield (Figure 4a). Furthermore, the in‐plane holes in PGCN/TNTs are also beneficial for rapid cross‐plane diffusion of mass, photogenerated carriers, and hydrogen, which dramatically accelerate the photocatalytic reaction in kinetics. Third, features including large accessible surface area and high hydrophilicity not only afford plentiful catalytically active centers, but also improve the reactant adsorption and diffusion process during the reaction. At last, H_2_O or dye molecules could experience the redox reactions around catalytic centers, generating H_2_ or being degraded.

**Figure 6:**
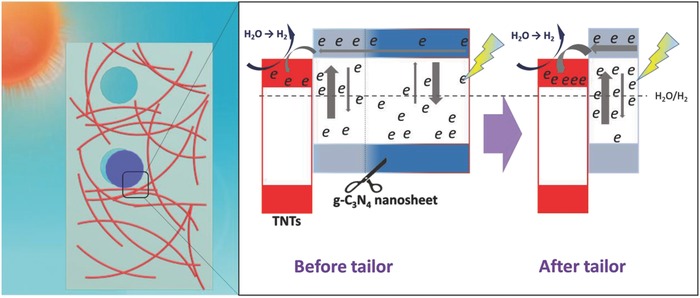
Schematic diagram showing the photocatalytic reaction over PGCN/TNTs as the photocatalyst under visible‐light irradiation.

In summary, we have designed and developed perforated ultralong TiO_2_ nanotube‐interlaced g‐C_3_N_4_ heterostructures (PGCN/TNTs) via a template‐tailoring process. Due to the cutting of redundant g‐C_3_N_4_ nanosheets and the construction of heterostructures, the migration distance of photoinduced charge transfer within PGCN/TNTs is shortened effectively. Thereby, intrinsic characteristics of easy charge recombination in g‐C_3_N_4_ nanosheets could be suppressed. The in‐plane holes and high hydrophilicity from PGCN/TNTs not only accelerate cross‐plane diffusion to dramatically promote photocatalytic reactions in kinetics, but also supply more catalytically active centers. The tailored heterostructures possessing a short electron transfer distance are highly favorable for electron–hole separation with a long lifetime. Accordingly, the obtained PGCN/TNTs exhibit a superior visible‐light H_2_‐generation activity of 1364 µmol h^−1^ g^−1^ (λ > 400 nm), which is ninefolds higher than that of bulk g‐C_3_N_4_ photocatalysts. This unique template‐tailoring strategy presents a significant improvement in reducing photoinduced electron–hole recombination for developing highly efficient visible‐light photocatalysts toward solar energy conversion.

## Conflict of Interest

The authors declare no conflict of interest.

## Supporting information

Supplementary
