# Supplementary material for: Tailoring TiO2 Nanotube‐Interlaced Graphite Carbon Nitride Nanosheets for Improving Visible‐Light‐Driven Photocatalytic Performance
Source: Adv Sci (Weinh). 2018 Apr 15;5(6):1700844. doi: 10.1002/advs.201700844 (PMC6010724; doi:10.1002/advs.201700844)
Supplement: Supplementary file 1 — Supplementary [file ADVS-5-1700844-s001.pdf]

Copyright WILEY-VCH Verlag GmbH & Co. KGaA, 69469 Weinheim, Germany, 2017.

ADVANCED SCIENCE
Open Access

Supporting Information

for *Adv. Sci.,* DOI: 10.1002/advs.201700844

Tailoring TiO_2 Nanotube-Interlaced Graphite Carbon Nitride Nanosheets for Improving Visible-Light-Driven Photocatalytic Performance

Yang Wang, Xueqin Liu, Cunchuan Zheng, Yinchang Li, Songru Jia, Zhen Li,* and Yanli Zhao**

Copyright WILEY-VCH Verlag GmbH & Co. KGaA, 69469 Weinheim, Germany, 2016.

Supporting Information

Tailoring TiO_2 Nanotube-Interlaced Graphite Carbon Nitrogen Nanosheets for Improving Visible-Light-Driven Photocatalytic Performance

Yang Wang, Xueqin Liu, Cunchuan Zheng, Yinchang Li, Songru Jia, Zhen Li,* Yanli Zhao**

1. Experimental section

1.1 Preparation of photocatalyst

Ultralong TiO_2 nanotubes (TNTs) were synthesized by a one-step hydrothermal process according to a previous report.[1] Typically, P25 powder (0.1 g) was dispersed into NaOH solution (15 mL, 10 M) with continuous stirring for 5 min, and then the solution was transferred into 25 mL Teflon-lined stainless steel autoclave with a magnetic stirrer. The autoclave was put inside a silicon oil bath on a hot plate and the reaction temperature was set at 130 °C for 24 h. After the reaction, the autoclave was taken out from oil bath and cooled to room temperature. The product, sodium titanate, was collected by centrifugation, washed with deionized water for several times to reach a pH value of 9. After that, the obtained precipitates were washed with HCl solution (0.1 M) and stirred at room temperature for overnight. The purified precipitates were rinsed with water several times followed by centrifugation. The obtained powders were dried in a vacuum drying chamber at 60 °C for 12 h. The final anatase TiO_2 nanotubes were obtained after annealing at 500 °C for 1 h.

The g-C_3N_4/TNTs were prepared by heating the mixture of TNTs and melamine. In a typical procedure, obtained TiO_2 nanotubes (100 mg) and melamine (6 g) were mixed in methanol (100 mL) followed by an ultrasonic treatment at 40 °C for 30 min. The mixture was stirred at room temperature for 4 h. The white solid was obtained through drying at 60 °C. Then, this white precursor in an alumina crucible was annealed at 550 °C inside a muffle furnace with aluminized paper to shelter from oxygen for 3 h. The final products were collected for use without further treatment.

The PGCN/TNTs were prepared by hydrolyzing bulk g-C_3N_4/TNTs in alkaline conditions. Briefly, g-C_3N_4/TNTs powder (1 g) was mixed with NaOH solution (20 mL, 3 M). The mixture was treated under ultra-sonication for 2 h. Then, PGCN/TNTs were dialyzed to remove excess NaOH using a membrane with molecular weight cutoff of 2000 Da (D306-50,

Biodesign Inc., U.S.A.) against water until reaching neutral pH. Finally, the solid PGCN/TNTs were collected with freeze drying.

1.2 Characterizations

The morphologies of the as-prepared samples were examined by field-emission scanning electron microscopy (FE-SEM, JSM-7600F, JOEL) at an acceleration voltage of 5 kV. The detailed structure analysis was conducted with transmission electron microscopy (TEM, JEOL JEM2100F) operating at 100 kV. Crystallographic information was collected by powder X-ray diffraction (XRD, D8 Advance diffractometer, Bruker) with Cu Kα radiation (λ = 1.5406 Å). The nitrogen adsorption/desorption isotherm was measured using a Micromeritics ASAP 2020 sorptometer. Thermogravimetric analysis (TGA Q500, TA) was carried out in air atmosphere from 40 to 900 °C with a ramping rate of 10 °C min^{-1}. Fourier transform infrared (FT-IR) spectra were recorded on a Nicolet 6700 spectrometer using the KBr pellet as the background. X-ray photoelectron spectroscopy (XPS) measurements were performed on an AXIS Ultra DLD (Kratos, USA) using monochromatic Al Kα X-ray source (anode HT = 15 kV) operating at a vacuum more than 10^{-7} Pa. Atomic force microscopy (AFM) was recorded by a Veeco Nanoscope IVa Multimode system. UV−vis diffuse reflection spectra (DRS) were recorded on a Shimadzu UV-2550 UV−vis spectrophotometer at normal temperature from 250 to 800 nm, in which fine $BaSO_4$ was used as the reflectance standard. The photoluminescence (PL) measurements were carried out using a Fluoromax 4P spectrofluorometer (Horiba) with a laser (λ = 380 nm) at room temperature. The time-resolved fluorescence measurements were recorded on an Edinburgh FLS980 at an excitation wavelength of 345 nm.

1.3 Photocatalytic measurements

In a typical experiment of water splitting for hydrogen evolution, photocatalyst (50 mg) and methanol (20 mL) serving as the sacrificial electron donor were added to ultrapure water (60 mL) under stirring. Then, H_2PtCl_6 aqueous solution was added as the precursor for the co-

catalyst Pt, which was in-situ photoreduced during the photocatalytic reaction (~3 wt% Pt). Pt serves as a co-catalyst in photocatalytic water splitting for its excellent electron extraction from the conductive band of the main catalyst. Finally, the sealed quartz tube was side-irradiated under visible light by using a 300 W xenon lamp equipped with a 400 nm cutoff filter. The reaction temperature was carefully maintained at room temperature. During the visible light irradiation, the evolved gas was collected at the given time intervals and analyzed with Shimadzu GC gas chromatography equipped with a thermal conductive detector (TCD) and high-purity Ar carrier gas.

The photocatalytic activity of each sample was evaluated in terms of the degradation of rhodamine B (RhB, 10 mg/L). The sample (20 mg) was added into a Pyrex photocatalytic reactor containing RhB solution (100 mL). A 300 W xenon lamp (with 400 nm cutoff filter) was used as visible light source. Prior to the irradiation, the suspension was stirred in the dark for 30 min to achieve the adsorption-desorption equilibrium. Aeration was performed using an air pump to ensure a constant supply of oxygen. At the given time intervals, the analytical samples were taken from the mixture and immediately centrifuged to remove the photocatalysts.

1.4 AQE measurements and wavelength dependent experiments

The apparent quantum efficiency (AQE) was detected using six LEDs at centered wavelengths of 380, 400, 420, 460, 500 and 460 nm. The photo intensity was confirmed by optical power meter (Advantest, Q8230). The irradiation area was controlled as 19.6 cm^2. The photocatalytic reaction was controlled as one hour. The AQY was calculated based on the following equation:

$$\text{AQE} = \frac{N_e}{N_p} \times 100\% = \frac{2 \times M \times N_A \times h \times c}{S \times P \times t \times \lambda} \times 100\%$$

where N_e is the amount of reaction electrons, N_p is the incident photons, M is the amount of H_2 molecules, N_A is Avogadro constant, h is the Planck constant, c is the speed of light, S is

the irradiation area, P is the intensity of the irradiation, t is the photoreaction time, and λ is the wavelength of the monochromatic light.

1.5 Photoelectrochemical testing

Electrochemistry impedance spectroscopy (EIS) and photocurrent intensity response measurements were performed on an electrochemical workstation (CHI600E, China) based on a conventional three electrode cell, where a sample-coated clean fluoride-tin oxide (FTO) glass, Pt wire and a saturated calomel electrode (SCE) were used as the working electrode, counter electrode and reference electrode, respectively. The aqueous solution of 0.2 M Na_2SO_4 purged with nitrogen gas was used as the electrolyte. The frequency range was from 0.01 Hz to 100 kHz, and the amplitude of the applied sine wave potential in each case was 5 mV for the EIS measurements. Incident light was obtained from a 300 W xenon lamp.

2. Figures

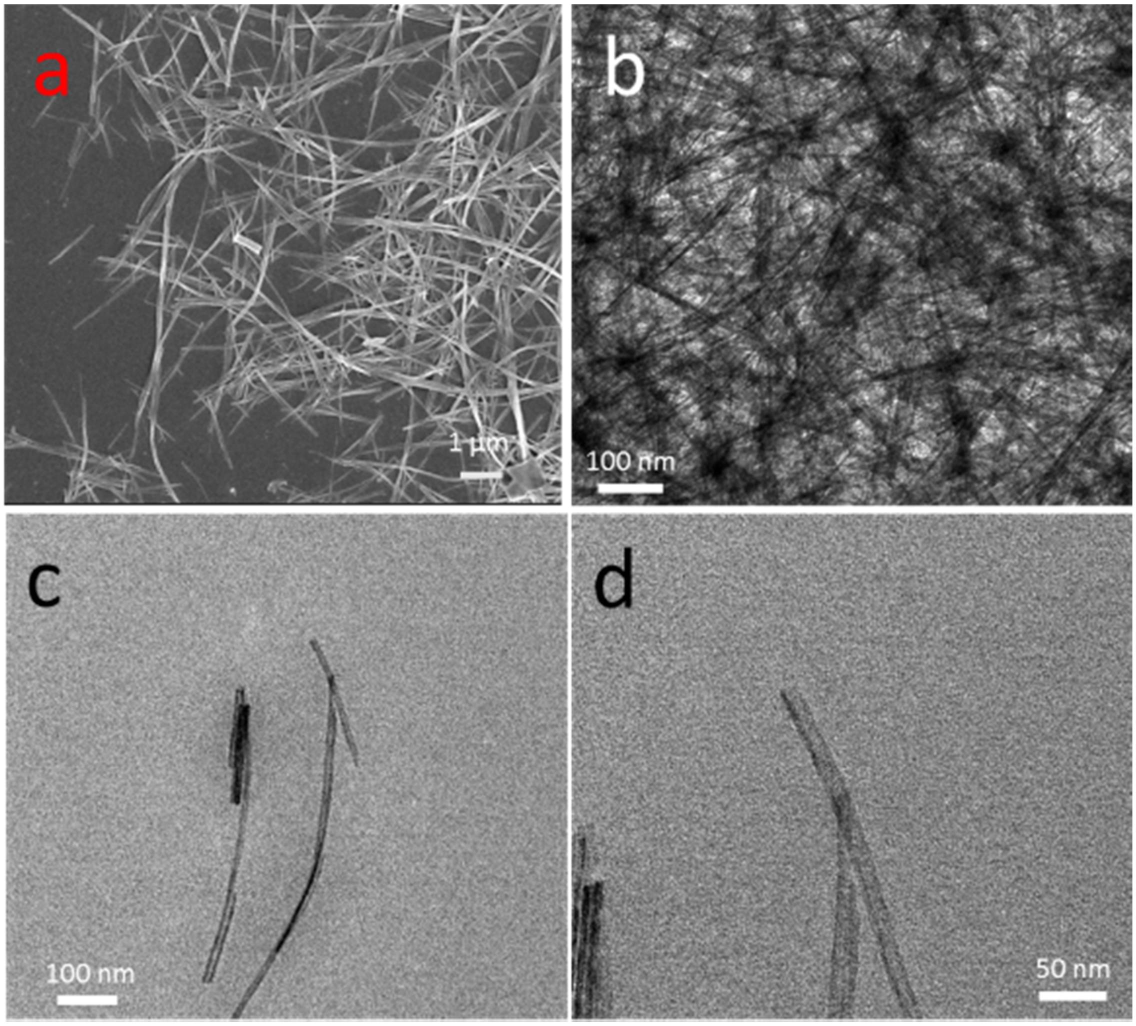

Figure S1. (a) SEM and (b-d) TEM images of ultra-long TiO_2 nanotubes.

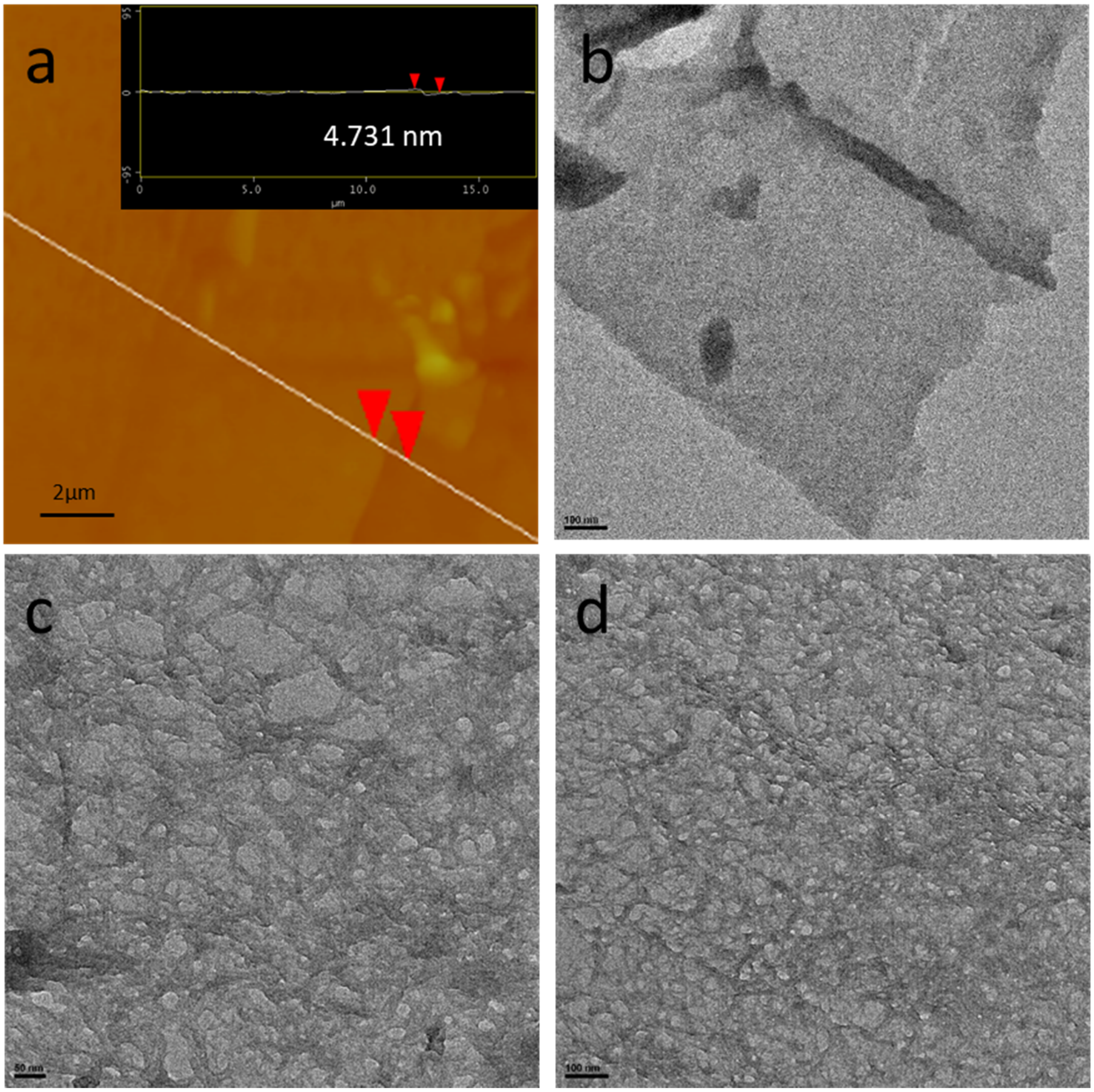

Figure S2. (a) AFM and (b) TEM images of exfoliated g-C_3N_4 nanosheets without sodium hydroxide. (c,d) TEM images of tailored g-C_3N_4 nanofibers with sodium hydroxide.

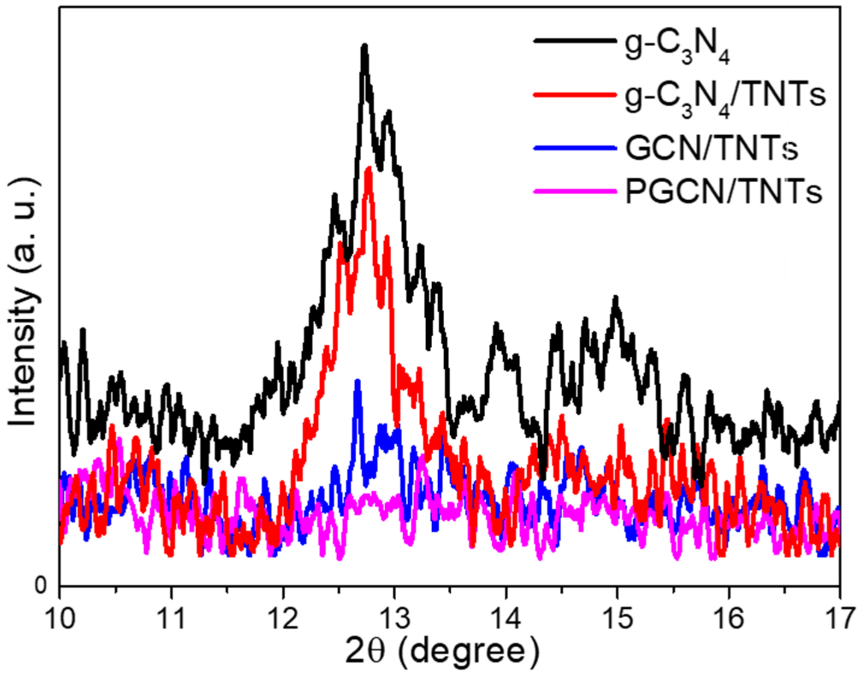

Figure S3. High-resolution powder XRD patterns of g-C_3N_4, g-C_3N_4/TNTs, GCN/TNTs, and PGCN/TNTs.

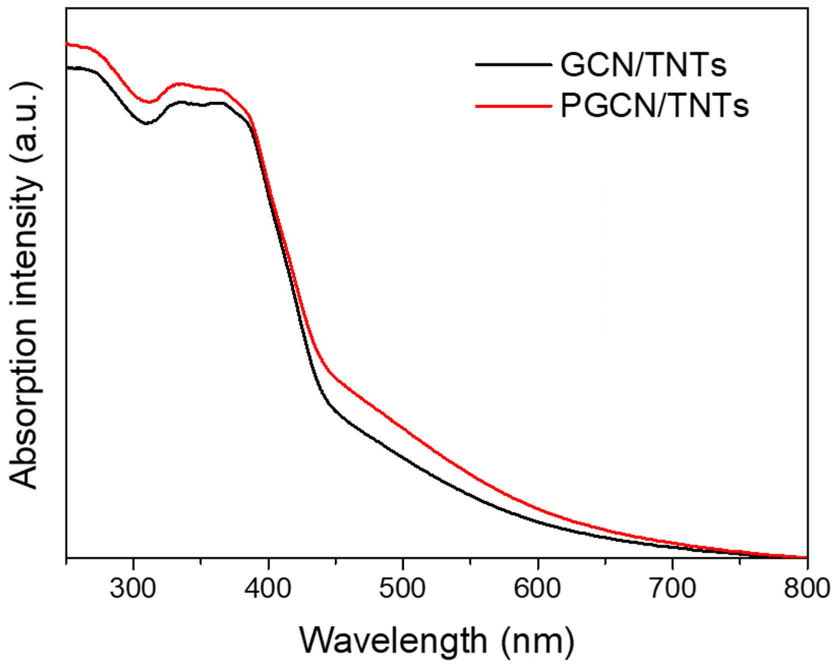

Figure S4. UV−vis diffuse reflectance spectra of GCN/TNTs and PGCN/TNTs.

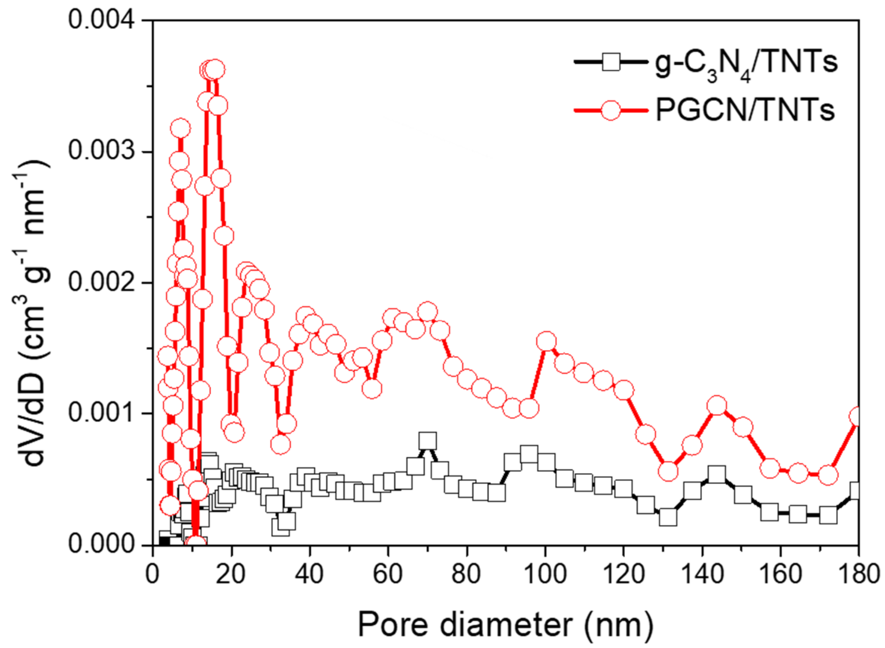

Figure S5. Pore size distribution plots of g-C_3N_4/TNTs and PGCN/TNTs.

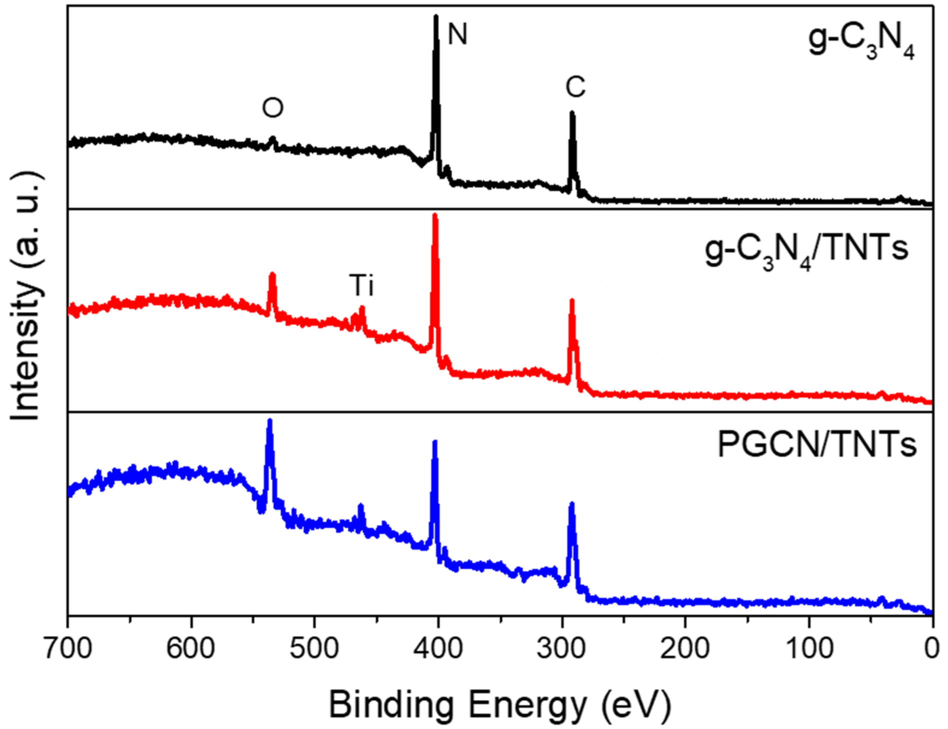

Figure S6. XPS spectra of g-C_3N_4, g-C_3N_4/TNTs and PGCN/TNTs.

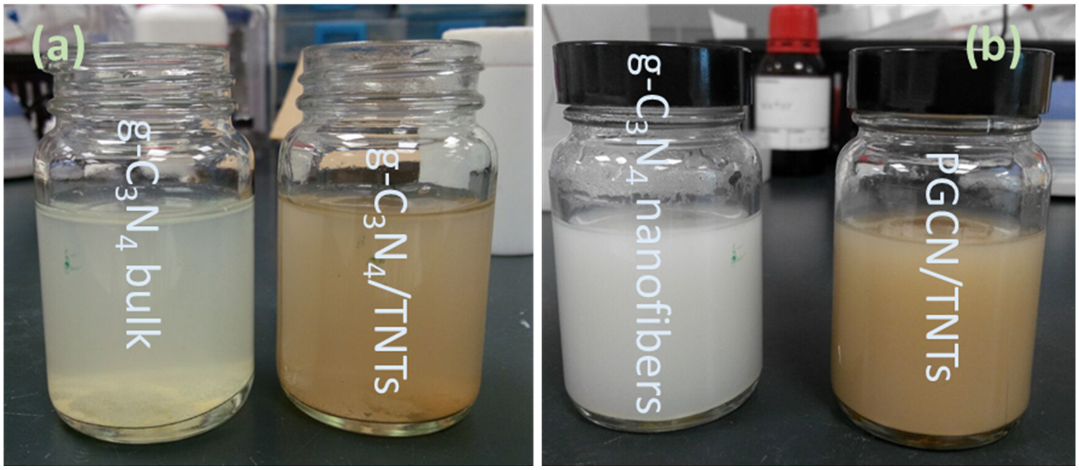

Figure S7. Dispersive ability comparison between (a) g-C_3N_4 and g-C_3N_4/TNTs, and (b) g-C_3N_4 nanofiber and PGCN/TNTs in aqueous solution.

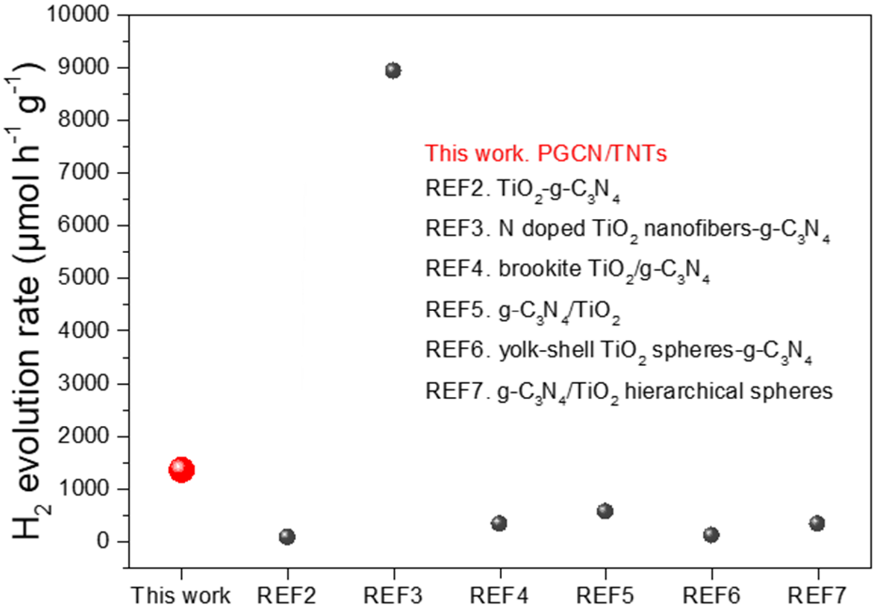

Figure S8. Summary of g-C_3N_4/TiO_2 based photocatalysts fabricated by melamine in terms of HER rate.

Table S1. Summaryof g-C_3N_4 based photocatalysts reported for AQE.

**Literature**	**AQE (%)**								
	380 nm	*400 nm*	*410 nm*	*420 nm*	*450 nm*	*460 nm*	*470 nm*	*500 nm*	*600 nm*
**[8]**		7.52	--	4.93	2.47	--	--	0.70	--
**[9]**		--	17.20	7.75	0.20	--	--	--	--
**[10]**	6.00	5.81	3.03	--	0.03	--	--	--	--
**[11]**	--	7.92	--	6.20	2.10	--	--	0	--
**[12]**	--	--	--	5.10	--	--	--	--	--
**This work**	10.47	8.21	--	6.32	--	2.33	--	1.04	0.32

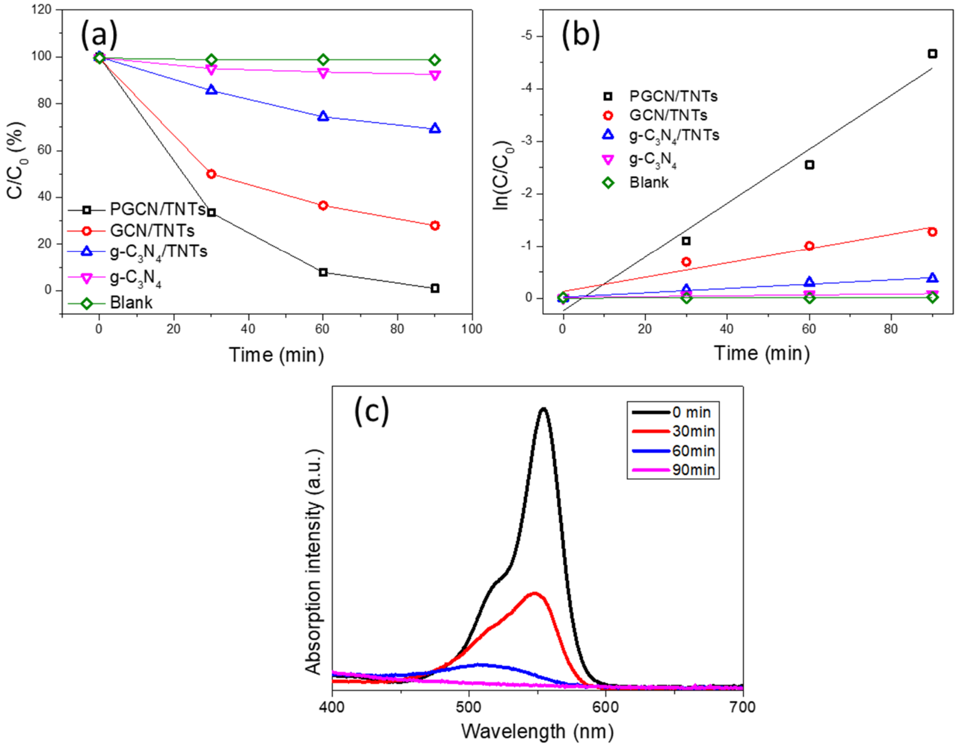

Figure S9. (a) Photocatalytic degradation of RhB over g-C_3N_4, g-C_3N_4/TNTs, GCN/TNTs and PGCN/TNTs under visible light irradiation. (b) Photocatalytic degradation curves of RhB solution under visible light irradiation in the control experiments. (c) UV-vis spectral changes of RhB over PGCN/TNTs.

The photocatalytic degradation of RhB dye was measured under visible light irradiation ($\lambda > 400$ nm). As presented in Figure S9a, nearly no RhB was photodegraded in the absence of photocatalysts. Bulk g-C_3N_4 only degrades about 5 % RhB within 90 min irradiation, while g-C_3N_4/TNTs, GCN/TNTs and PGCN/TNTs all exhibit much higher RhB degradation capacity than bulk g-C_3N_4. PGCN/TNTs were able to completely degrade RhB within 90 min irradiation, which are better than g-C_3N_4, g-C_3N_4/TNTs and GCN/TNTs. The RhB degradation obeys the first-order kinetics, that is, $ln(C_0/C) = kt$, where C_0 and C are the concentration at the time zero and the concentration at time t for RhB respectively, and k is the degradation reaction rate constant. The k value of PGCN/TNTs is 0.051 min^{-1}, which is about 3.6 and 12.8 times higher than that of GCN/TNTs and g-C_3N_4/TNTs, respectively (Figure S9b).

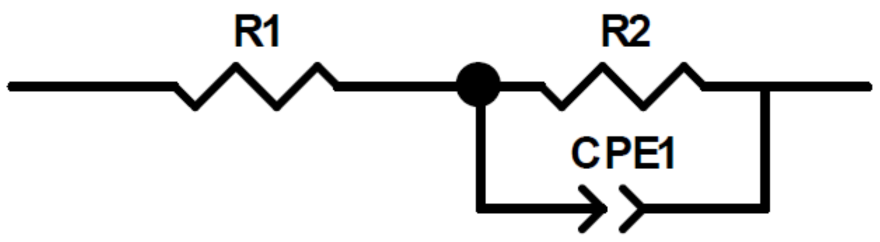

Figure S10. Transmission line model used for fitting the impedance spectra. R1 is the series resistance, including the sheet resistance of FTO and the contact resistance; R2 is the resistance for the electron transfer; CPE1 is double-layer capacitance at solution contact interface.

Table S2. EIS results of the four films.

Samples	Rs (Ω)	R1(Ω)	CPE1-T	CPE1-P
**g-C_3N_4**	47.37	3.44×10^{5}	5.9×10^{-5}	0.787
**g-C_3N_4/TNTs**	72.54	2.65×10^{5}	5.4×10^{-5}	0.784
**GCN/TNTs**	140.43	1.65×10^{5}	5.2×10^{-5}	0.812
**PGCN/TNTs**	75.88	1.04×10^{5}	4.47×10^{-5}	0.853

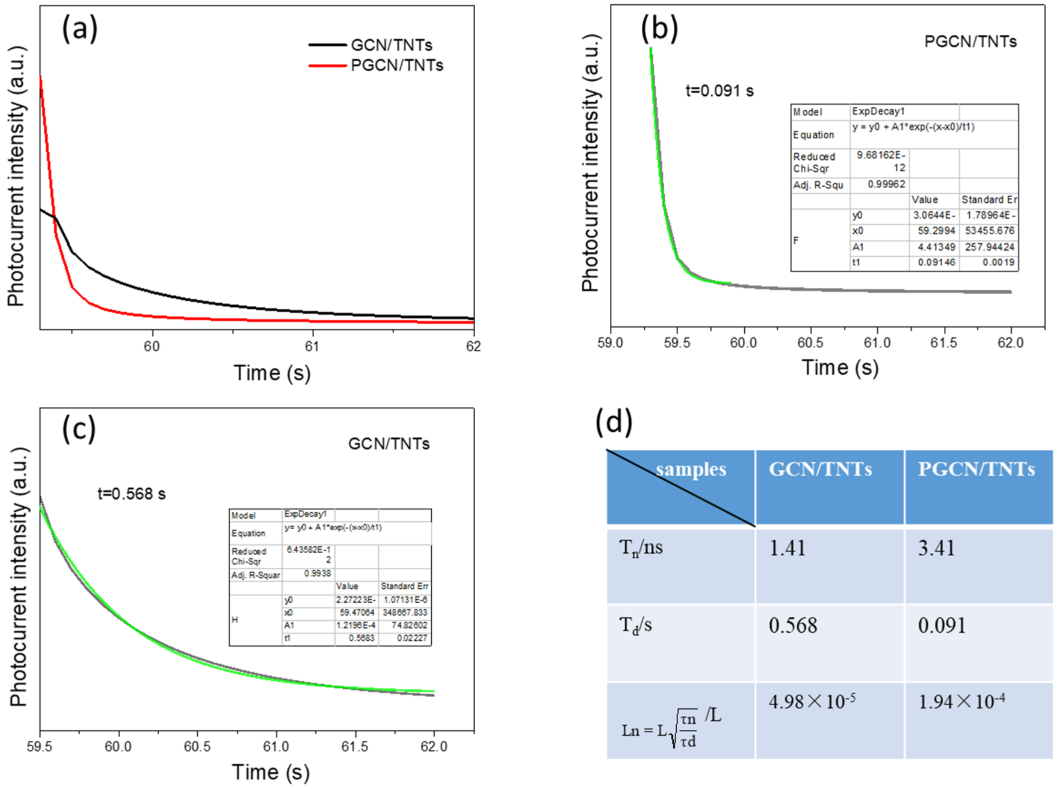

Figure S11. (a-d) Photocurrent decay and electron transport length calculation of GCN/TNTs and PGCN/TNTs.

Figure S11a shows the enlarged view of transient photocurrent from GCN/TNTs and PGCN/TNTs. It was found that the photocurrent decaying rate of PGCN/TNTs is faster than that of GCN/TNTs. The electron transfer time (τ_d) can be fitted to one major single

exponential decay process,[13] and it is 0.568 and 0.091 s, respectively. By combining the results of PL lifetime (τ_n), the electron diffusion length (L_n) was obtained according to the following calculation formula:

$$L_n = L\sqrt{\frac{\tau_n}{\tau_d}}$$

where L is the length of the semiconductor. It turned out that the L_n of PGCN/TNTs is 2.9 times higher than that of GCN/TNTs.

References

[1] Y. Tang, Y. Zhang, J. Deng, J. Wei, H. L. Tam, B. K. Chandran, Z. Dong, Z. Chen, X. Chen, *Adv. Mater.* **2014**, *26*, 6111-6118.

[2] H. Yan, H. Yang, *J. Alloy. Compd.* **2011**, *509*, 26-29.

[3] C. Han, Y. Wang, Y. Lei, B. Wang, N. Wu, Q. Shi, Q. Li, *Nano Res.* **2015**, *8*, 1199-1209.

[4] Y. Zang, L. Li, Y. Xu, Y. Zuo, G. Li, *J. Mater. Chem. A* **2014**, *2*, 15774-15780.

[5] J. Wang, J. Huang, H. Xie, A. Qu, *Int. J. Hydrogen Energ.* **2014**, *39*, 6354-6363.

[6] Z. Jiang, C. Zhu, W. Wan, K. Qian, J. Xie, *J. Mater. Chem. A* **2016**, *4*, 1806-1818.

[7] J. Ma, X. Tan, T. Yu, X. Li, *Int. J. Hydrogen Energ.* **2016**, *41*, 3877-3887.

[8] J. Am. Chem. Soc. 2017, 139, 3021−3026W. Che, W. Cheng, T. Yao, F. Tang, W. Liu, H. Su, Y. Huang, Q. Liu, J. Liu, F. Hu, Z. Pan, Z. Sun, S. Wei, *J. Am. Chem. Soc.* **2017**, *139*, 3021−3026.

[9] H. Ou, L. Lin, Y.Zheng, P. Yang, Y. Fang, X. Wang, *Adv. Mater.* **2017**, *29*, 1700008.

[10] H. Ou, P. Yang, L. Lin, M. Anpo, X. Wang, *Angew. Chem. Int. Ed.* **2017**, *56*, 10905–10910.

[11] Y. Kofuji, Y. Isobe, Y. Shiraishi, H. Sakamoto, S. Tanaka, S. Ichikawa, T. Hirai, *J. Am. Chem. Soc.* **2016**, *138*, 10019−10025.

[12] Q. Han, B. Wang, J. Gao, Z. Cheng, Y. Zhao, Z. Zhang, L. Qu, *ACS Nano* **2016**, *10*, 2745–2751.

[13] Q. Wang, J. E. Moser, M. Grätzel, *J. Phys. Chem. B* **2005**, *109*, 14945-14953.
